# Only When It Feels Good: Specific Cat Vocalizations Other Than Meowing

**DOI:** 10.3390/ani9110878

**Published:** 2019-10-29

**Authors:** Jaciana Luzia Fermo, Maria Alice Schnaider, Adelaide Hercília Pescatori Silva, Carla Forte Maiolino Molento

**Affiliations:** 1 Animal Welfare Laboratory, Federal University of Paraná, Curitiba 80035-050, Paraná, Brazil; jacianafermo@gmail.com (J.L.F.); maschnaider@yahoo.com.br (M.A.S.); 2 Department of Literature and Linguistics, Federal University of Paraná, Curitiba 80035-050, Paraná, Brazil; adelaidehpsilva@gmail.com

**Keywords:** cat behavior, *Felis catus*, phonetics, welfare

## Abstract

**Simple Summary:**

Among carnivore animals, domestic cats are those with the most extensive vocal repertoire. This is due to their social organization, nocturnal activity and long period of contact between the mother and the offspring. In order to identify vocalizations other than meowing in two different situations, a study was performed with 74 cats divided into two groups, one associated with a pleasant situation and another with an aversive situation. Only the group exposed to the positive stimulus of being offered a favorite snack produced specific vocalizations other than meowing: recognition or trill, squeak, purring and chatter. During the aversive situation of car transport, no vocalization other than meowing was observed. The present study indicates the relevance of applying the study of vocalizations to determine the state of emotional valence in cats.

**Abstract:**

Our objective was to identify and characterize the types of vocalization other than meowing (VOM) in two contexts, a pleasant and an aversive situation, and to study the effect of the sex of the animal. A total of 74 cats (32 tom cats and 42 queens) living in the city of Curitiba, Brazil, participated in the study; in total, 68 (29 tom cats and 39 queens) were divided into two groups according to the stimulus they were exposed to: either a pleasant situation (PS), when they were offered a snack, or an aversive situation (AS), with the simulation of a car transport event. The other six animals (three tom cats and three queens) participated in both situations. Only the PS group presented VOM; of the 40 PS animals, 14 presented VOM, mostly acknowledgment or trill and squeak. No correlation was observed between vocalization and cat sex (*p* = 0.08; Pearson’s Chi-Square). Results show that VOM is exclusively associated with positive situations, suggesting that these vocalizations may be relevant for understanding the valence of cat emotional state. Further studies are warranted to advance knowledge on other VOMs and on the generalization of our findings to other situations.

## 1. Introduction

Communication plays a central role in various aspects of animal life, such as during breeding interactions, territorial defense, parental care and anti-predator behavior in many species. Vocalization is the active generation of sounds with the use of specific organs, which propagate through acoustic signals that transmit a wide range of information about the communicator, including his or her emotional, motivational and physiological states [1,2]. A number of studies used vocalization as an analytical tool to assess animal welfare [3,4,5,6,7,8,9], using non-invasive monitoring systems. The vocalization events captured may then be studied with the aid of programs that perform a detailed acoustic analysis of sound waves, employing parameters such as frequency and amplitude of the signals [2], in order to classify the different types of vocalization.

A better understanding of how animals communicate their feelings is fundamental for all aspects of animal welfare, which is an ever increasing societal demand. As an essential mode for communicating feelings and emotions in the human species, we are particularly prone to understanding oral expressions, provided they occur in a manner that we have decodified. In the context of vocalizations as indicators of animal welfare, cats (*Felis catus*) are of special interest. They have lived with humans for over 10,000 years and have become one of the most popular pet species in the world, with over 600 million individuals [10,11]. Their repertoire of vocalization is more extensive as compared to other members of the Order Carnivorae [12,13], which may be explained by their social organization, nocturnal activity and long period of contact between the mother and her offspring [12]. There are studies that report up to 17 distinct cat vocal signals and there is ongoing research to understand the meaning of these sounds especially when they are aimed at humans [14].

Vocal responses may prove to be useful tools for motor, perceptual, motivational and social development investigations in the cat [15]. Cats can express affiliative feelings during human–animal or mother–kitten interactions, as well as fear and aggression in hostile situations through vocalization and body posture [16]. Cat vocalizations are generally divided into three main categories [14,17,18]: murmur patterns, vowel patterns and forced intensity patterns. The murmur vocalization corresponds to the emission of sounds produced with the mouth closed. Examples of these sounds are purring, acknowledgment or trill, calling and grunting [19]. The vowel patterns are formed with the initially open mouth that closes gradually [19]; examples of this pattern are meowing, which occurs in a variety of situations during friendly cat–human interactions [12], more often than in cat–cat interactions [13]. The squeak, which is a loud nasal hoarse sound [20,21], is also classified as a vowel pattern of cat vocalization. Patterns of forced intensity are sounds that express aggressive emotional states and are produced with an open mouth in a relatively constant position [19,20]. Grunting, howling, growling, hissing and spitting are sounds related to aggression of various kinds [22]. Another type of forced intensity sound is the chatter, which is believed to be emitted when cats visualize prey in an attempt to mimic their sound [21]. Even though there is clear importance of vocalization in cats, there are few phonetic studies on this species and these report results of critically limited numbers of individuals, types of vocalization and methods [21].

The objective of this study was to identify and characterize the types of vocalization other than meowing (VOM) in two different contexts, a pleasant and an aversive situation, and to study the effect of the sex of the animal, in order to improve human understanding of cat communication, especially regarding emotional valence. Our hypotheses are that specific types of VOM are coherently and consistently related to either negative or positive emotional valence, since the prevalence of VOM in cats suggests they present relevant functions to the animals, and that female cats are more frequent VOM emitters than male cats, due to the important role mothers play in raising kittens and the many vocalizations involved in mother–kitten interactions.

## 2. Material and Methods

### 2.1. Animals

The project was conducted with the collaboration of cat guardians, residents in the city of Curitiba, Paraná, Southern Brazil, from 30 November 2017 to 22 March 2018. We first tested 223 mixed-bred domestic cats, exposing each cat to both an aversive (AS) and a pleasant (PS) situation. Cats were then selected using the criterion of a vocalization rate equal to or greater than five events during the recording time in each situation. This criterion resulted in the selection of 74 cats (29 males and 45 females). Among them, 68 (26 males and 42 females) reached the selection criterion only in one situation, either the AS or the PS. The other six animals (three males and three females) presented the required vocalization rate when exposed to both scenarios. This allowed for the inclusion of 40 cats per group; cats in the AS group were from four months to 14 years old, and 24 were spayed females and 16 were neutered males; the 40 cats in the PS group were between one and 14 years old, and 24 were spayed females and 16 were neutered males.

Individual recordings were made with a digital camera (Sony Cyber Shot DSC-W610 14.1 megapixels, Sony do Brasil Ltda, Rua Werner Von Siemens, 111, Lapa, CEP: 05069-010, São Paulo, SP), following the instructions proposed by Yeon et al. [16], adapted to the use of a single camera. AS was produced by placing the cat in a carrying case, which was placed in the back seat of a car, where the cat was taken for a short trip in the absence of its guardian. The recording was initiated after the car started moving, but the evaluation started after the first vocal signal of the meow. The camera was attached to the carrying case with dimensions of 55 cm in height, 52 cm in width and 71 cm in length. The PS was produced by guardians offering each cat a portion of approximately 85 g of their favorite snack in their home environment. The snack was delivered to the cat only after the vocalization of the animal. In this case, the camera was placed on a tripod, facing the animal, at a distance of approximately 1.5 m from the guardian and cat pair, set and turned on prior to the start of the test session to allow the animal to adapt to the presence of the camera. Recording began shortly before the snack was offered and the evaluation was performed from the first meow vocal signal.

### 2.2. Vocal Measures

The audio were analyzed continuously for a period of 3 min starting from the first vocal sound emitted by the animal—for this, we considered any vocal sound, including meowing. To perform detailed vocalization analyses, audio were separated from videos using the Audacity software (version 2.1.3). The vocalization acoustic signals, which were captured at a sampling rate of 44.1 kHz and quantized at 16 bits, were stored in.wav format files on a computer Dell Inspiron 5458, with which the acoustic analyses were performed. Audio were carefully analyzed and categorized by one author—aided by interactions with other two authors for the first analyses, and the website Meowsic [23]. For the confirmation of purr detection, two authors were directly involved, since this type of vocalization is out of Praat software frequency range of detection and was very subtle in the audio recordings. Additionally, acoustic analysis of VOM events was performed with the Praat software, version 5.3.55, developed by Paul Boersma and David Weenink, at the Institute of Phonetic Sciences at the University of Amsterdam. Other authors such as Yeon et al. [16] have previously used Praat to analyze animal vocalizations.

The vocalization variables were calculated separately for each individual. Measurements taken were identification, duration, fundamental frequency (f0) and intensity of VOM.

### 2.3. Statistical Analysis

The comparison between treatment groups, AS and PS, was obtained through descriptive statistics, since VOM values for AS were zero. To verify the relationship between the specific VOM emissions and the sex of the cat, Pearson’s Chi-Square statistical test in Microsoft Excel was used, considering a significance level of *p* < 0.05.

### 2.4. Ethical Note

This experiment was approved by the Ethics Committee on Animal Use (Comissão de Ética no Uso de Animais - CEUA) of the Sector of Agrarian Sciences of the Federal University of Paraná—Brazil, during the session of 2 June 2017, registered under protocol number 055/2017.

## 3. Results and Discussion

Vocalizations other than meowing were completely absent when cats were exposed to AS, which was a major finding of this work: when facing the specific AS studied, the only type of vocalization cats emitted was meowing. Thus, the types of VOM emitted by cats when exposed to the specific PS studied seem to be exclusively related to feelings of positive valence. Of the 40 animals (24 females and 16 males) exposed to the PS, 14 animals presented VOM (35.0%), which were recognition or trilling, purr, squeak and chatter types of vocalization. Of the 14 animals, 11 were female and three were male cats; no correlation was observed between vocalization and cat sex (*p* = 0.08). The literature provides reasons for hypothesizing a difference between sex for cat vocalization, since females play an important role in raising kittens, an activity highly related to vocal communication [12]. Thus, considering available knowledge and the statistical result observed for sex comparison (*p* = 0.08), further studies involving more animals in PS seem warranted to better understand the relationship between cat vocalization and sex. The group of cats that did present VOM did not include kittens or juveniles.

The duration, fundamental frequency and intensity of the different types of vocalization identified in this study, as well as the number and sex of cats who emitted them, are shown in Table 1. 

The acknowledgment/trill was emitted by six females and had an average duration of 0.32 s, an f0 of 454.92 Hz and intensity of 56.58 dB. The male feline emitted this sound, which lasted 0.34 s, had no f0 captured by the Praat program and an intensity of 52.0 dB. In the study by Schötz [21], the trill was the most common sound after the meow, presented with a duration of 0.51 if f0 of 533 Hz, and the wave image also presented the same characteristics. The trill has already been reported in the context of the friendly approach of cats to familiar people, an expression of strong bonding to their guardians, meaning a form of recognition [12,19].

The squeak sound was emitted by four females with an average duration of 0.36 s, f0 of 440.47 Hz and intensity of 56.24 dB. A male feline produced this sound, with a duration of 0.81 s, the f0 of 509.38 Hz and intensity of 61.06 dB. There are no publications of the waveform of this sound and, because it is part of the category of vocal patterns, it has been compared to the sound of meowing. No f0 was detected for this vocalization, possibly due to the distance between the camera and animal. Schötz [21] described that the meowing f0 ranges from 221 to 1185 Hz, with a mean duration of 0.42 s. According to Schötz [14], a squeak is a raspy, nasal, high-pitched and often short meow-like call, sometimes not ending with a closing mouth and indicates a friendly request, coinciding with the context of our PS.

The purr was emitted by two males with an average duration of 2.51 s and intensity of 45.78 dB. This sound was also emitted by a female with a duration of 1.10 s and an intensity of 47.77 dB. The purr f0 was not detected by the Praat program, probably because it is likely to be below 50 Hz and may be also due to the presence of background noise. According to Bradshaw et al. [13], the purr can last 2–700 s with f0 of 25–30 Hz, there is no intensity information, and it is usually associated with contact situations. Humans often interpret purring as if the cat were happy, but some cats also purr at feeding time, requesting their food [24]. The acoustic qualities of this vocalization are difficult to ignore and are a sign of care and attention solicitude, which is usually reinforced by the guardians [13]; Thus, it seems coherent that cats in the PS emitted those sounds. However, purring requires further research, especially designed for this vocalization. It is extremely subtle and our experimental design and equipment composed a setting where the possibility of undetected purring may not be excluded.

The chatter was produced by a cat in this study with a duration of 1.05 s and an intensity of 50.49 dB; no f0 was detected for this vocalization, possibly due to the distance between the camera and the animal. Schötz [21] reported a chatter average duration of 0.74 s, f0 of 400–600 Hz, with no description of its intensity. This type of vocalization may be considered an imitation of the sounds emitted by prey in order to deceive them, as for example when cats observe birds through a window [13]. In this study, a cat produced this vocalization trying to reach the snack, which for the animal may mean the search for food, with putative hunting components.

## 4. Conclusions

For the first time, specific vocalizations other than meowing were identified as exclusively emitted in a situation of positive emotional valence: acknowledgement or thrill, squeaking, purring and chatter, emitted by cats when exposed to a pleasant situation of receiving their favorite snack. No difference was reported between sex; however, this issue warrants further studies with a higher number of individuals. As no vocalization other than meowing was observed during the aversive situation of car transport, the vocalization types studied seem highly relevant for understanding the valence of cat emotional state. Overall, this research indicates the relevance of applying the study of sounds other than meowing for the comprehension of the responses of cats when facing different situations and emotional states, additionally advancing knowledge regarding their characterization. Further studies are warranted to advance knowledge regarding the generalization of our findings to other pleasant and aversive situations.

## Figures and Tables

**Table 1:** Average and standard deviation for duration, fundamental frequency (f0) and intensity of the different types of vocalizations other than meowing (VOM) observed during the pleasant situation (PS) by sex of the animal, according to the analyses of 74 cats facing positive and aversive situations; only cats in the positive situations expressed VOM.

Types of Vocalization	Number of Cats	Sex	Duration (Seconds)	Fundamental Frequency (Hertz)	Intensity (Decibel)
**Trill**	1	Male	0.34	-	52.0
	6	Female	0.32 ± 0.19	454.92 ± 89.44	56.58 ± 5.46
**Squeak**	1	Male	0.81	509.38	61.06
	4	Female	0.36 ± 0.28	440.47 ± 80.29	56.24 ± 7.70
**Purr**	2	Male	2.51 ± 2.12	-	45.78 ± 1.47
	1	Female	1.10	-	47.77
**Chatter**	0	Male	-	-	-
	1	Female	1.05	-	50.49

## References

[1] Grandin T. (1998). The feasibility of using vocalization scoring as an indicator of poor welfare during slaughter. Appl. Anim. Behav. Sci..

[2] Friel M., Kunc H.P., Griffin K., Asher L., Collins L.M. (2016). Acoustic signalling reflects personality in a social mammal. R. Soc. Open Sci..

[3] Weary D., Fraser D. (1997). Vocal response of piglet to weaning: Effect of piglet age. Appl. Anim. Behav. Sci..

[4] Burgdof J., Panksepp J., Moskal J.R. (2011). Frequency-modulated 50 kHz ultrasonic vocalizations: A tool for uncovering the molecular substrates of positive affect. Neurosci. Biobehav. Rev..

[5] Matthews S.G., Miller A.L., Clapp J., Plötz T., Kyriazakis I. (2016). Early detection of health and welfare compromises through automated detection of behavioural changes in pigs. Vet. J..

[6] Cordeiro A.F.D.S., Nääs I.D.A., Baracho M.D.S., Jacob F.G., Moura D.J.D. (2018). The use of vocalization signals to estimate the level of pain in piglets. Eng. Agric..

[7] Urrutia A., Martínez-Byer S., Szenczi P., Hudson R., Bánszegi O. (2019). Stable individual differences in vocalisation and motor activity during acute stress in the domestic cat. Behav. Process..

[8] Nicastro N., Owren M.J. (2003). Classification of domestic cat (*Felis catus*) vocalizations by naive and experienced human listeners. J. Comp. Psychol..

[9] Nicastro N. (2002). Acoustic correlates of human responses to domestic cat (*Felis catus*) vocalizations. J. Acoust. Soc. Am..

[10] Turner D.C., Bateson P. (2000). The Domestic Cat: The Biology of Its Behaviour.

[11] Driscoll C.A., Juliet C.B., Andrew C.K., Stephen J.O.B. (2009). The taming of the cat. Sci. Am..

[12] Bradshaw J.W.S. (1992). The Behaviour of the Domestic Cat.

[13] Bradshaw J.W.S., Beaumont C., Turner D.C., Bateson P. (2000). The signaling repertoire of the domestic cat and its undomesticated relatives. the Domestic Cat—The Biology of Its Behaviour.

[14] Schötz S., Van de Weijer J., Eklund R. (2017). Phonetic Characteristics of Domestic Cat Vocalisations. the 1st International Workshop on Vocal Interactivity in-and-between Humans, Animals and Robots.

[15] Brown K.A., Buchwald J.S., Johnson J.R., Mikolich D.J. (1978). Vocalization in the cat and kitten. Dev. Psychobiol..

[16] Yeon S.C., Kim Y.K., Park S.J., Lee S.S., Suh Y.L., Houpt K.A., Chang H.H., Lee H.C., Yang B.G., Lee H.J. (2011). Differences between vocalization Evoked by social stimuli in feral cats and house cats. Behav. Process..

[17] Moelk M. (1944). Vocalizing in the House-Cat: A Phonetic and Functional Study. Am. J. Psychol..

[18] Crowell-Davis S.L., Curtis T.M., Knowles R.J. (2004). Social organization in the cat: A modern understanding. J. Feline Med. Surg..

[19] Beaver B.V. (2005). Comportamento Felino—Um Guia Para Veterinários.

[20] Little S.E. (2015). O Gato: Medicina Interna.

[21] Schötz S. (2012). A phonetic pilot study of vocalisations in three cats. Proc. Fon..

[22] Rochlitz I. (2005). The Welfare for Cats.

[23] Meowsic. http://vr.humlab.lu.se/projects/meowsic/catvoc.html.

[24] McComb K., Taylor A.M., Wilson C., Charlton B.D. (2009). The cry embedded within the purr. Curr. Biol..

